# Association between hypertriglyceridemic-waist phenotype and non-alcoholic fatty liver disease: a general population-based study

**DOI:** 10.1186/s12944-022-01660-8

**Published:** 2022-06-02

**Authors:** Ming Yang, Yaqin Xu, Chong Hu, Shuhua Zhang, Maobin Kuang, Yang Zou

**Affiliations:** 1 Department of Cardiology, Jiangxi Provincial People’s Hospital, Nanchang, 330006 Jiangxi China; 2 Department of Respiratory and Critical Care Medicine, The Second Affiliated Hospital of Nanchang University, Nanchang, 330006 Jiangxi China; 3 Department of Gastroenterology, Jiangxi Provincial People’s Hospital, Nanchang, 330006 Jiangxi China; 4 Jiangxi Cardiovascular Research Institute, Jiangxi Provincial People’s Hospital, Nanchang, 330006 Jiangxi China

**Keywords:** Non-alcoholic fatty liver disease, Hypertriglyceridemic-waist phenotype, HTGW, General population, Triglyceride, Waist circumference

## Abstract

**Background:**

Hypertriglyceridemic-waist (HTGW) phenotype has been proposed as a practical tool for screening the risk of cardiovascular diseases and glycemic metabolic disease. This study sought to investigate the relationship between HTGW phenotype and non-alcoholic fatty liver disease (NAFLD).

**Methods:**

A total of 14,251 subjects who took part in health screening were enrolled in the study and NAFLD was diagnosed by abdominal ultrasound. According to triglyceride (TG) and waist circumference, the study population was divided into four phenotypes, in which HTGW phenotype was defined as TG ≥ 1.7 mmol/L and male waist circumference ≥ 90 cm or female waist circumference ≥ 80 cm. Multivariate logistic regression analysis was used to evaluate the relationship between HTGW phenotype and NAFLD.

**Results:**

In the current study, 2.43% of the subjects had HTGW phenotype, while the prevalence of NAFLD in subjects with HTGW phenotype was 77.81%. After full adjustment for covariates, compared with people with normal waist circumference and TG levels, the risk of NAFLD in people with normal TG levels but enlarged waist circumference increased by 39% [OR:1.39, 95%CI: 1.15, 1.68], in people with normal waist circumference but elevated TG levels increased by 96% [OR:1.96, 95%CI: 1.65, 2.33], and in subjects with HTGW phenotype increased by 160% [OR:2.60, 95%CI: 1.88, 3.58]. Additionally, further analysis suggested that there were significant interactions between age, height, BMI and NAFLD risk associated with TGW phenotypes. Receiver operating characteristic curves analysis suggested that the combination of TG and waist circumference further improved the diagnostic value for NAFLD.

**Conclusions:**

HTGW phenotype is associated with NAFLD risk in the general population, which may be a novel and accessible indicator for NAFLD screening.

**Supplementary Information:**

The online version contains supplementary material available at 10.1186/s12944-022-01660-8.

## Background

Non-alcoholic fatty liver disease (NAFLD) is one of the chronic non-communicable diseases with the highest incidence in the world, mainly manifesting as hepatic steatosis [1]. Besides marked hepatic steatosis, NAFLD can also cause a wide range of metabolic disorders, significantly increase the risk of type 2 diabetes, cardiovascular disease and chronic kidney disease, and negatively affect health-related quality of life in the patients [2–4]. It was reported that the current prevalence of NAFLD in the world is about 25.24% (27.37% in Asia) [5], and the public awareness rate is about 6.3–18% [6, 7]. Compared with diabetes and hypertension (prevalence 9.3 and 31.1%, awareness rates 30–74.8% and 32.3–67%) [8–11], NAFLD has a very low awareness rate despite a high prevalence. This dangerous situation was also explicitly mentioned in a recent statement of the NAFLD Consensus Consortium and advocated the development of a NAFLD public health roadmap to advance the global public health agenda for NAFLD [12]. As a health worker, to promote the early formulation of NAFLD public health strategy and improve the awareness rate of NAFLD, our current study aims to find a simple, low-cost, and effective method for NAFLD screening in a large population.

The hypertriglyceridemic waist (HTGW) phenotype is a physical feature classified by triglycerides (TG) and waist circumference which is characterized by enlarged waist circumference and elevated TG levels. HTGW phenotype and its concept were first noticed and studied by Lemieux et al. [13]. According to their early description, they found that men with HTGW phenotype will have an increased risk of atherosclerosis, and most people with HTGW phenotype had an obvious metabolic disorder. In this context, a growing number of scholars have conducted an in-depth analysis of the HTGW phenotype. Many studies have shown that the HTGW phenotype was not only associated with coronary artery disease, but also closely related to pancreatitis, metabolic syndrome, diabetes, pre-diabetes, hyperuricemia, ischemic stroke, chronic nephropathy, and visceral obesity [14–21]. Also, in the recent study by Blackburn et al., it was found that the HTGW phenotype had the same ability to identify adverse metabolic characteristics as the National Cholesterol Education Program-Adult Treatment Panel III standard and International Diabetes Federation (IDF) standard [22]. All these pieces of evidence suggested that the HTGW phenotype may be an adverse phenotype for metabolic-related diseases. At present, several studies have specifically assessed the association between HTGW phenotype and NAFLD in children and adolescents, premenopausal and postmenopausal women, and overweight/obese people [23–25]. However, the association between HTGW phenotype and the risk of NAFLD in the general population is not clear. Therefore, through the secondary analysis of the large longitudinal cohort of NAGALA, this study aims to further evaluate the performance of the HTGW phenotype as a screening tool for the risk of NAFLD in the general population.

## Methods

The datasets used in this study have been publicly available in the Dryad data repository (source data uploaded by Professor Okamura) and can be accessed through 10.5061/dryad.1n6c4 [26]. According to the user terms of the Dryad database, the Dryad dataset can be used for academic research, but not for commercial purposes.

### Study population and design

The current study is a secondary analysis of population data from the NAGALA cohort, whose study design has been published elsewhere [27]. In short, the NAGALA cohort was established by Murakami Memorial Hospital in Japan in 1994 and has continued to the present day, enlisting adults who attended health screening at the hospital’s physical examination center and conducting a series of epidemiological studies mainly on diabetes and NAFLD. The focus of this study was to investigate the performance of the HTGW phenotype in assessing the risk of NAFLD. For this objective, we extracted data from 20,944 subjects in the NAGALA cohort from 1994 to 2015, and excluded subjects with the following characteristics: (1) excessive drinking (*n* = 1952, male ≥210 g/w or female ≥140 g/w) [28]; (2) suffering from viral/alcoholic hepatitis or diabetes or impaired fasting glucose (*n* = 1547; according to the self-reported diagnosis or baseline survey found abnormal blood glucose); (3) baseline information missing (*n* = 873); and (4) receiving medication at baseline (*n* = 2321). Informed consent for data use has been described in previous studies with the subject’s authorization [27]. Additionally, the current research protocol has been approved by the Institutional Review Committee of Jiangxi Provincial People’s Hospital (review No: 2021–066), and the entire research process follows the Declaration of Helsinki.

### Anthropometric and laboratory measurements

The general data were collected by trained medical staff using standardized health questionnaires. The recorded information comprised socio-demographic characteristics (age and sex), living habits (smoking, drinking, and habit of exercise), disease history (diabetes and liver disease), and general body measurements (height, weight, waist circumference, and blood pressure). Body mass index (BMI) was calculated as weight/height^2^. Having an exercise habit was defined as regular participation in any type of sports more than once a week. Drinking status: During the baseline visit, the subjects’ weekly alcohol intake was evaluated and classified, in which the weekly alcohol consumption of less than 40 g was defined as not drinking or small drinking, the weekly alcohol consumption of 40-139 g was defined as light drinking, and the weekly alcohol consumption of 140-209 g was defined as moderate drinking. Smoking status: During the baseline interview, the subjects were divided into three groups: non-smoking, past smoking, and current smoking by inquiring the subjects about their smoking history.

After fasting overnight, blood samples were collected from each subject. The biochemical indexes of aspartate aminotransferase (AST), fasting plasma glucose (FPG), TG, gamma-glutamyl transferase (GGT), hemoglobin A1c (HbA1c), alanine aminotransferase (ALT), high-density lipoprotein cholesterol (HDL-C) and total cholesterol (TC) were determined by standard experimental methods.

### Diagnosis of NAFLD

NAFLD was scored and diagnosed by experienced gastroenterologists based on the four abdominal ultrasonographic features of vascular blurring, hepatorenal echo contrast, deep attenuation, and liver brightness without knowing the subjects’ other examination results [29].

### Statistical analysis

Based on the IDF standard [30], the subjects were divided into four triglyceride waist circumference (TGW) phenotypes (Table 1), and the baseline characteristics of the subjects were summarized according to different TGW phenotypes. Before the inter-group comparison, the distribution pattern of continuous variables was judged by the QQ plot, and the variables were described as mean (standard deviation: SD) and median (quartile1–3) respectively according to the distribution pattern. The differences between groups of continuous data with a normal or approximate normal distribution were compared by one-way ANOVA test, and Tukey’s HSD test was used as a post hoc test. The differences between groups of continuous data with skewness distribution were compared by the Kruskal-Wallis H test, and the Steel Dwass test was used as a post hoc test. Categorical variables described as n (%), comparison between groups using chi-square test.

**Table 1:** Definitions of the four triglyceride waist phenotypes

Triglyceride waist phenotypes	Definition
NTNW	Normal TG level (≤150 mg/dL (1.7 mmol/L)) and Normal waist circumference (< 90 cm in males or < 80 cm in females)
NTEW	Normal TG level (≤150 mg/dL (1.7 mmol/L)) and Enlarged waist circumference (≥90 cm in males or ≥ 80 cm in females);
ETNW	Elevated TG level (> 150 mg/dL (1.7 mmol/L)) and Normal waist circumference (< 90 cm in males or < 80 cm in females)
HTGW	Elevated TG level (> 150 mg/dL (1.7 mmol/L)) and Enlarged waist circumference (≥90 cm in males or ≥ 80 cm in females)

Note: *HTGW *hypertriglyceridemic–waist, *TG *triglyceride, *WC *waist circumference

To systematically account for potential confounders, after collinearity diagnosis of covariates (Supplementary Table 1) [31], we tested the effects of four different TGW phenotypes on NAFLD in four stepwise adjusted multivariable logistic regression models [32], with the results expressed as odds ratios (OR) and 95% confidence intervals (CI). In addition, considering the obvious physical differences between the sexes, we also evaluated the effect of TGW phenotypes on NAFLD in men and women separately in four multivariate logical regression models. In model 1, BMI, age, and sex were adjusted; Model 2 additionally adjusted habit of exercise and height on the basis of model 1; Model 3 further adjusted SBP, drinking status, and smoking status; Model 4 continued to adjust HbA1c, FPG, TC, and HDL-C based on model 3. We also exploratory evaluated the associations between different TGW phenotypes and NAFLD in different populations by logistic regression (based on model 4), and used the likelihood ratio test to detect the interaction between TGW phenotypes and covariables. Finally, to further verify the diagnostic performance of TG, waist circumference, and the combination of the two indexes in NAFLD, we also constructed receiver operating characteristic curves (ROC) and calculated the corresponding area under the curve (AUC). Delong test was used to check the difference between the index after the combination of TG and waist circumference and the single waist circumference, TG.

All analyses were conducted using the statistical software R language (version 3.4.3) and Empower (R) (version 2.0). For all analyses, *P* values < 0.05 (two-sided) were considered statistically significant.

## Results

### Baseline characteristics of subjects with different TGW phenotypes

A total of 14,251 subjects who participated in health screening were included in this study, and they were divided into groups according to different TGW phenotypes. As shown in Table 2, in this study, 11,483 subjects (80.58%) had normal TG and waist circumference (NTNW), 914 subjects (6.41%) had elevated TG and normal waist circumference (ETNW), 1507 subjects (10.57%) had normal TG and enlarged waist circumference (NTEW), and 347 subjects (2.43%) had both increased TG and waist circumference (HTGW). The prevalence of NAFLD in the four phenotypes was 10.29, 46.17, 42.00 and 77.81%, respectively. Compared with subjects with the NTNW phenotype, subjects with the other three phenotypes generally had lower HDL-C levels, higher height, ALT, age, HbA1c, TG, GGT, BMI, TC, weight, AST, waist circumference, FPG, and higher blood pressure. Furthermore, in the ETNW group and HTGW group, the proportion of males was significantly increased, and the number of subjects with smoking and drinking habits was significantly higher.

**Table 2:** Characteristics of the participants in four triglyceride waist phenotypes

Variables	NTNW	ETNW	NTEW	HTGW
No. of subjects	11,483 (80.58%)	914 (6.41%)	1507 (10.57%)	347 (2.43%)
Age, years	42.00 (36.00–49.00)	45.00 (39.00–52.00)	45.00 (39.00–52.00)*^#^	43.00 (38.00–50.50)^##^
Sex				
Female	5731 (49.91%)	86 (9.41%)	941 (62.44%)	82 (23.63%)
Male	5752 (50.09%)	828 (90.59%)	566 (37.56%)	265 (76.37%)
Weight, kg	57.66 (9.86)	67.12 (8.12)	71.36 (13.13)	79.96 (11.43)
Height, cm	164.45 (8.41)	168.59 (7.08)	164.14 (8.93)**	169.27 (8.56)^#^
BMI, kg/m^2^	21.21 (2.44)	23.57 (2.12)	26.33 (3.24)	27.81 (2.72)
Waist circumference, cm	73.60 (7.37)	81.23 (5.21)	88.87 (7.09)	93.30 (6.02)
ALT, U/L	16.00 (12.00–21.00)	24.00 (18.00–32.00)	19.00 (14.00–27.00)	30.00 (21.00–45.00)
AST, U/L	17.00 (14.00–20.00)	20.00 (16.00–24.00)	18.00 (15.00–23.00)*	22.00 (18.00–29.00)
GGT, U/L	14.00 (11.00–19.00)	24.00 (17.00–35.00)	16.00 (12.00–24.00)	28.00 (20.00–40.50)
HDL-C, mmol/L	1.52 (0.40)	1.07 (0.25)	1.35 (0.34)	1.06 (0.22)^#^
TC, mmol/L	5.03 (0.84)	5.67 (0.88)	5.30 (0.83)	5.87 (0.86)
TG, mmol/L	0.65 (0.45–0.94)	2.13 (1.86–2.59)	0.87 (0.60–1.17)	2.17 (1.90–2.65)^#^
FPG, mmol/L	5.10 (0.41)	5.38 (0.37)	5.27 (0.40)	5.43 (0.35)^#^
HbA1c, %	5.15 (0.31)	5.23 (0.34)	5.31 (0.33)	5.32 (0.34)^##^
SBP, mmHg	111.96 (14.03)	120.16 (13.85)	121.72 (15.53)	129.00 (15.44)
DBP, mmHg	69.76 (9.86)	76.32 (9.88)	76.02 (10.77)	81.13 (10.59)
Habit of exercise	2096 (18.25%)	148 (16.19%)^*^	187 (12.41%)	39 (11.24%)
Drinking status				
Non or small	9538 (83.06%)	687 (75.16%)	1308 (86.79%)	272 (78.39%)
Light	1407 (12.25%)	140 (15.32%)	155 (10.29%)	56 (16.14%)
Moderate	538 (4.69%)	87 (9.52%)	44 (2.92%)	19 (5.48%)
Smoking status				
Non	7219 (62.87%)	337 (36.87%)	1029 (68.28%)	161 (46.40%)
Past	2019 (17.58%)	239 (26.15%)	241 (15.99%)	60 (17.29%)
Current	2245 (19.55%)	338 (36.98%)	237 (15.73%)	126 (36.31%)
NAFLD	1182 (10.29%)	422 (46.17%)	633 (42.00%)	270 (77.81%)

*Abbreviations*: *BMI* Body mass index, *ALT* Alanine aminotransferase, *AST* Aspartate aminotransferase, *GGT* Gamma-glutamyl transferase, *HDL-C* High-density lipoprotein cholesterol, *TC* Total cholesterol, *TG* Triglyceride, *HbA1c* Hemoglobin A1c, *FPG* Fasting plasma glucose, *SBP* Systolic blood pressure, *DBP* Diastolic blood pressure, *NAFLD* Non-alcoholic fatty liver disease, *NTNW* Normal TG and WC, *ETNW* Elevated TG and normal WC, *NTEW* Normal TG and enlarged WC, *HTGW* Hypertriglyceridemic-waist

Values were expressed as mean (SD) or medians (quartile interval) or n (%)

Since there are significant differences between almost all groups after pairwise comparison, it is difficult to mark all the groups with differences in the table. Therefore, in Table 2, we only marked the values that showed no significant difference after pairwise comparison

*P > 0.05 vs ETNW, **P > 0.05 vs NTNW, ^#^*P* > 0.05 vs ETNW, ^##^*P* > 0.05 vs NTEW. Other variables with no special mark on the upper right corner had *P* values < 0.05 after pair-wise comparison

### TGW phenotypes and NAFLD

The relationship between TGW phenotypes and NAFLD is shown in Table 3. Whether in men, women, or the whole population, subjects with ETNW, NTEW and HTGW phenotypes had a significantly higher risk of developing NAFLD than subjects with NTNW phenotype, and subjects with HTGW phenotype had a significantly higher risk of developing NAFLD than subjects with NTNW, NTEW and ETNW phenotypes (Model 1–4). Additionally, it is worth noting that in the fully adjusted model (Model 4), compared with the NTNW phenotype, the risk of NAFLD in female subjects with HTGW phenotype increased by 3.49 times (OR: 4.49, 95%CI: 2.52–8.01), while in male and the overall study population, the NAFLD risk of subjects with HTGW phenotype increased by 0.85 times (OR: 1.85, 95%CI: 1.28–2.69) and 1.6 times (OR: 2.60, 95%CI: 1.88–3.58) respectively.

**Table 3:** Logistic regression analysis assessing the associations between the HTGW phenotype and NAFLD

	Odds ratios (95% confidence interval)			
	NTNW	ETNW	NTEW	HTGW
**All**				
Model 1	Ref*	3.11 (2.66, 3.65)*	1.42 (1.18, 1.70)*	3.81 (2.79, 5.20)
Model 2	Ref*	3.09 (2.63, 3.62)*	1.45 (1.20, 1.74)*	3.88 (2.84, 5.32)
Model 3	Ref*	3.12 (2.66, 3.67)*	1.47 (1.22, 1.77)*	4.04 (2.94, 5.54)
Model 4	Ref*	1.96 (1.65, 2.33)*	1.39 (1.15, 1.68)*	2.60 (1.88, 3.58)
**Women**				
Model 1	Ref*	3.75 (2.07, 6.79)*	1.86 (1.38, 2.50)*	9.48 (5.38, 16.71)
Model 2	Ref*	3.73 (2.06, 6.76)*	1.94 (1.43, 2.63)*	9.81 (5.55, 17.34)
Model 3	Ref*	3.55 (1.95, 6.47)*	1.91 (1.41, 2.60)*	9.64 (5.43, 17.10)
Model 4	Ref*	1.74 (0.94, 3.22)*	1.42 (1.09, 1.84)*	4.49 (2.52, 8.01)
**Men**				
Model 1	Ref*	3.03 (2.57, 3.58)*	1.26 (0.99, 1.59)*	2.46 (1.73, 3.49)
Model 2	Ref*	3.00 (2.54, 3.54)*	1.27 (1.00, 1.62)*	2.47 (1.73, 3.51)
Model 3	Ref*	3.05 (2.58, 3.61)*	1.32 (1.03, 1.69)*	2.64 (1.84, 3.79)
Model 4	Ref*	1.94 (1.62, 2.34)*	1.26 (0.98, 1.62)*	1.85 (1.28, 2.69)

Abbreviations as in Table 2; Model 1 adjusted for sex, age and BMI; Model 2 adjusted for model 1 plus habit of exercise and height. Model 3 adjusted for model 2 plus SBP, smoking status and drinking status. Model 4 adjusted for model 3 plus HbA1c, FPG, TC and HDL-C

*P < 0.05 compared with HTGW phenotype

In the sex subgroup, the sex was not adjusted in the model

### Subgroup analysis

Table 4 shows the associations between TGW phenotypes and NAFLD in different populations. As can be seen, among all covariates, only age, height and BMI were found to have an interaction effect on NAFLD risk associated with the TGW phenotype. Compared with young people with NTNW phenotype, any other TGW phenotype populations in the same age groups and higher age groups had a higher risk of NAFLD. Compared with non-obese people with the NTNW phenotype, both non-obese and obese people with other TGW phenotypes had a higher risk of NAFLD. Compared with people with short stature with NTNW phenotype, taller people with NTNW phenotype had lower NAFLD risk, while people with other TGW phenotypes had relatively higher NAFLD risk.

**Table 4:** Effect size of TGW phenotypes on NAFLD in exploratory subgroups

	adjusted Odds ratios (95% confidence interval)				
	NTNW	ETNW	NTEW	HTGW	*P for interaction*
Age (years)					0.0425
18–29	Ref	6.42 (0.93, 44.30)	1.13 (0.29, 4.45)	__^a^	
30–44	2.05 (1.05, 4.00)	5.35 (2.66, 10.74)	3.01 (1.50, 6.06)	5.96 (2.70, 13.15)	
45–59	2.50 (1.28, 4.89)	3.76 (1.86, 7.59)	3.37 (1.68, 6.76)	5.45 (2.45, 12.13)	
≥ 60	1.52 (0.73, 3.18)	1.73 (0.68, 4.37)	2.56 (1.10, 5.97)	7.73 (1.79, 33.35)	
Height (m)					0.0098
Low	Ref	1.35 (0.96, 1.91)	1.42 (1.11, 1.83)	4.12 (2.36, 7.20)	
High	0.93 (0.78, 1.12)	2.03 (1.60, 2.58)	1.25 (0.96, 1.61)	1.93 (1.31, 2.84)	
Weight (kg)					0.1046
Low	Ref	1.50 (0.90, 2.50)	2.05 (1.38, 3.05)	2.88 (0.98, 8.48)	
High	1.22 (0.98, 1.52)	2.43 (1.86, 3.18)	1.59 (1.19, 2.13)	3.16 (2.11, 4.74)	
BMI (kg/m^2^)					0.0147
< 25	Ref	2.12 (1.75, 2.57)	4.14 (3.20, 5.35)	5.86 (3.01, 11.42)	
≥ 25	3.59 (3.01, 4.27)	6.69 (4.87, 9.21)	8.79 (7.37, 10.48)	14.34 (10.29,20.00)	
Habit of exercise					0.4137
No	Ref	2.01 (1.67, 2.43)	1.33 (1.09, 1.63)	2.60 (1.85, 3.66)	
Yes	0.87 (0.73, 1.05)	1.48 (1.01, 2.17)	1.57 (1.06, 2.31)	2.22 (0.97, 5.10)	
ALT (U/L)					0.1011
< 40	Ref	1.82 (1.51, 2.20)	1.34 (1.10, 1.64)	2.62 (1.83, 3.74)	
≥ 40	4.74 (3.69, 6.11)	12.25 (7.26, 20.64)	5.64 (3.49, 9.11)	5.53 (2.85, 10.71)	
AST (U/L)					0.7095
< 40	Ref	1.93 (1.62, 2.30)	1.39 (1.14, 1.68)	2.54 (1.83, 3.52)	
≥ 40	2.91 (1.72, 4.94)	11.57 (3.10, 43.13)	3.45 (1.37, 8.71)	6.54 (1.44, 29.76)	
GGT (U/L)					0.2751
< 40	Ref	2.05 (1.69, 2.48)	1.44 (1.18, 1.75)	2.56 (1.80, 3.66)	
≥ 40	1.56 (1.24, 1.96)	2.14 (1.52, 3.01)	1.64 (1.04, 2.59)	3.57 (1.85, 6.89)	
HDL-C (mmol/L)					0.1834
< 1.03	Ref	2.35 (1.80, 3.06)	1.01 (0.71, 1.45)	2.66 (1.66, 4.25)	
≥ 1.03	0.60 (0.50, 0.72)	1.35 (1.02, 1.78)	0.90 (0.70, 1.16)	1.96 (1.25, 3.09)	
TC (mmol/L)					0.0915
< 5.2	Ref	2.12 (1.59, 2.83)	1.67 (1.31, 2.14)	2.18 (1.21, 3.92)	
≥ 5.2	1.66 (1.44, 1.91)	3.25 (2.65, 3.99)	1.95 (1.53, 2.48)	4.71 (3.26, 6.81)	
FPG (mmol/L)					0.4621
< 5.6	Ref	2.11 (1.72, 2.60)	1.42 (1.15, 1.75)	2.91 (1.99, 4.26)	
≥ 5.6	1.43 (1.22, 1.68)	2.42 (1.84, 3.18)	1.87 (1.38, 2.52)	2.88 (1.69, 4.90)	
HbA1c (mmol/L)					0.3226
Low	Ref	2.05 (1.58, 2.64)	1.21 (0.91, 1.61)	2.22 (1.37, 3.61)	
High	1.39 (1.21, 1.60)	2.60 (2.06, 3.27)	2.13 (1.69, 2.69)	4.02 (2.67, 6.06)	
SBP (mmHg)					0.3960
< 140	Ref	2.01 (1.68, 2.40)	1.42 (1.17, 1.73)	2.46 (1.75, 3.45)	
≥ 140	1.52 (1.14, 2.02)	2.37 (1.37, 4.10)	1.61 (1.05, 2.45)	5.87 (2.34, 14.71)	
SBP (mmHg)					0.7907
< 90	Ref	2.00 (1.67, 2.39)	1.41 (1.16, 1.71)	2.56 (1.82, 3.61)	
≥ 90	1.18 (0.86, 1.62)	1.83 (1.08, 3.11)	1.36 (0.85, 2.18)	3.11 (1.36, 7.11)	
Drinking status					0.0879
Non or small	Ref	1.88 (1.55, 2.29)	1.33 (1.08, 1.63)	3.35 (2.28, 4.92)	
Light	0.58 (0.47, 0.72)	1.21 (0.82, 1.78)	0.96 (0.64, 1.46)	0.75 (0.40, 1.39)	
Moderate	0.56 (0.41, 0.75)	1.36 (0.83, 2.23)	1.16 (0.60, 2.26)	0.92 (0.33, 2.60)	
Smoking status					0.4716
Non	Ref	1.87 (1.42, 2.46)	1.48 (1.17, 1.86)	3.51 (2.17, 5.68)	
Past	0.97 (0.82, 1.16)	2.06 (1.51, 2.81)	1.31 (0.93, 1.86)	1.47 (0.76, 2.83)	
Current	0.74 (0.62, 0.89)	1.42 (1.08, 1.87)	0.92 (0.65, 1.30)	1.81 (1.10, 2.98)	

^a^The model failed because of the small sample size. Abbreviations as in Table 2;

Adjusted for sex, age, BMI, habits of exercise, height, smoking status, drinking status, SBP, HbA1c, FPG, TC and HDL-C

In each case, the model is not adjusted for the stratification variable

### ROC analysis

Table 5 shows the AUC of parameters of TG, waist circumference and the combination of the two which were used to identify the NAFLD, in which TG was 0.7969 and waist circumference was 0.8610, and the AUC after their combination further increased to 0.8803. Compared with TG and waist circumference alone, the combination of the two can enhance the diagnostic ability for NAFLD.

**Table 5:** Areas under the receiver operating characteristic curves of the Indicators to detect nonalcoholic fatty liver disease

	AUC	95%CI low	95%CI upp	Specificity	Sensitivity
**All cohort**					
TG*	0.7969	0.7877	0.8061	0.6799	0.7698
Waist circumference*	0.8610	0.8539	0.8681	0.7571	0.8085
TG + Waist circumference	0.8803	0.8737	0.8868	0.7374	0.8588

*Abbreviations*: *AUC* Area under the curve; other abbreviations as in Table 1. *P < 0.0001, compared with TG + Waist circumference

## Discussion

In this large epidemiological study of 14,251 people in the general population, we found that compared with people with normal waist circumference and TG levels, hypertriglyceridemia alone or increased waist circumference alone significantly increased the risk of NAFLD, while when hypertriglyceridemia coexisted with elevated waist circumference the risk of NAFLD would further increase.

It is well known that hypertriglyceridemia and central obesity are important risk factors for NAFLD [33, 34]. In the case of hypertriglyceridemia or increased waist circumference, insulin resistance is a common metabolic change, which leads to excessive releasing of fatty acids from adipose tissue and up-regulating the transcription of genes that promote liver ab initio adipogenesis. These reactions can result in liver steatosis [35]. In the current study, we further confirmed in a large sample that either hypertriglyceridemia alone or central obesity significantly increased the risk of NAFLD [ETNW: OR: 1.96, 95%CI: 1.65, 2.33; NTEW: OR: 1.39, 95%CI: 1.15, 1.68]. Additionally, it is worth mentioning that the OR value associated with NAFLD risk in subjects with ETNW phenotype was higher than that in subjects with NTEW phenotype.

HTGW is a special state that comprises both enlarged waist circumference and elevated TG levels [13]. This special phenotype may be a sign of lipid spillover caused by relative defects in adipose tissue [36]. Past studies have provided a great deal of evidence that this particular phenotype was closely related to a variety of metabolic diseases [14–22]. However, at present, the research on the association between HTGW phenotype and NAFLD is limited, and there are still significant differences in several similar studies. In the earliest investigation and analysis of 962 children and adolescents aged 6–18 years old by Hosseini et al., it was proposed for the first time that the phenotype of HTGW may be related to NAFLD [23]. Subsequently, similar findings were observed in two Chinese studies, among which Liu et al. specially evaluated 1779 premenopausal and postmenopausal women [24], while Zhou et al. chose overweight/obese people [25]. Considering that these similar studies had some particularity in population selections and their sample size was relatively small, this study further explored the relationship between HTGW phenotype and NAFLD in the general population on the basis of a larger sample. According to the current research results, we found that when central obesity and hypertriglyceridemia appeared simultaneously, the risk of NAFLD in the general population would further increase (comparing with NTNW, NTEW, and ETNW; all *P* < 0.05). This conclusion is also applicable to other metabolism-related diseases [14–21].

We also analyzed the associations between TGW phenotypes and NAFLD in men and women. In both sexes, the risk pattern between TGW phenotypes and NAFLD was consistent with that of the whole population. However, it is worth noting that compared with male subjects, female subjects with HTGW phenotype had a relatively higher risk of NAFLD. Similarly, this gender stratification finding has also been reported in TGW phenotypes related studies conducted by Ren and Chen et al., in which Ren et al. assessed the gender differences in the association between diabetes and HTGW phenotype [16], while Chen et al. analyzed the gender differences between hyperuricemia and HTGW phenotype [18]. In addition, some different results have been shown in other studies related to TGW phenotypes: In a follow-up study of 4081 subjects with stroke, Wang et al. indicated that the HTGW phenotype was only associated with future stroke events in women [19], while in another study on risk assessment of chronic kidney disease, only male HTGW phenotype was associated with chronic kidney disease [20]. In general, there are some differences in the HTGW phenotype across disease risk assessments, and more studies are needed to further validate these results. According to the current results of gender stratification, women with HTGW phenotype should pay more attention to screening for NAFLD.

The current study also exploratory analyzed the interactions between all covariates and TGW phenotypes-related NAFLD risk. From the results, there were significant interactions between age, height, BMI and TGW phenotypes, among which the elderly people, short stature people, and overweight/obese people with HTGW phenotype seemed to have the highest risk of NAFLD. Generally speaking, aging, short stature, and overweight/obesity often indicate potential adverse metabolic characteristics [37–39]. Therefore, these people should pay more attention to NAFLD screening.

The high prevalence of HTGW phenotype in some common diseases also requires some special attention. According to the published literature data, about 38.9–42.1% of the subjects with HTGW phenotype had pre-diabetes [17, 40, 41], 22.7–38.10% had diabetes [16, 40–42], 25.9% had hyperuricemia [18], and 17–28.1% had chronic kidney disease [20, 43]. Additionally, according to Lemieux et al., more than 80% of male subjects with the HTGW phenotype had abnormal metabolic characteristics that cause atherosclerosis [13]. These findings conveyed an intuitive message that the HTGW phenotype was a very adverse metabolic feature. In the current study, we found that nearly 80% of subjects with the HTGW phenotype had NAFLD. Given the high prevalence of the HTGW phenotype in multiple metabolic diseases, we suggest that the HTGW phenotype should be incorporated into screening programs for NAFLD and other metabolic diseases. Furthermore, it is worth noting that the HTGW phenotype is closely related to cardiovascular risk, and cardiovascular events are the main cause of mortality and morbidity in patients with NAFLD [2, 13]. Therefore, we speculate that the HTGW phenotype may be used to predict cardiovascular events in patients with NAFLD. It needs to be confirmed in further research in the future.

### Study strength and limitation

The biggest strength of the current study is that it has been confirmed that the HTGW phenotype was associated with an increased risk of NAFLD in the general population in a large sample. These results further expanded the current research evidence and provided useful data for the application of the HTGW phenotype for NAFLD screening in the general population.

Limitation: (1) The design adopted in the current study was cross-sectional, so whether there was a causal correlation between HTGW phenotype and NAFLD needs to be further confirmed in longitudinal studies. In addition, there was a lack of follow-up information on cardiovascular disease in the current dataset, so we cannot further evaluate the associations between TGW phenotypes and future cardiovascular events in the NAFLD population. (2) At present, the gold standard of NAFLD diagnosis is still based on the results of liver biopsy, but in the current study, NAFLD was diagnosed by abdominal ultrasound, which would inevitably lead to missing some patients with mild hepatic steatosis [44]. (3) Although a large number of confounding factors have been corrected in the current research, there were still some unmeasured or unmeasurable confounding factors that would partially affect the results. (4) The correlation between HTGW phenotype and liver fibrosis could not be further analyzed in the current study due to the absence of some parameters used to calculate non-invasive fibrosis scores in the public dataset currently analyzed.

## Conclusion

In summary, the general population with ETNW, NTEW, and HTGW phenotypes had a significantly increased risk of NAFLD compared with the population with the NTNW phenotype, and those with the HTGW phenotype had the highest risk of NAFLD. The findings of this study provided evidence of the association between HTGW phenotype and NAFLD in the general population, and these findings may have important public health implications for the early diagnosis and intervention of NAFLD.

## Supplementary Information

**Additional file 1.**

## Data Availability

The data used in this study have been uploaded to the “Dryad” database by Professor Okamura et al.

## Abbreviations

HTGW: Hypertriglyceridemic-waist
NAFLD: Non-alcoholic fatty liver disease
TG: Triglyceride
IDF: International Diabetes Federation
BMI: Body mass index
AST: Aspartate aminotransferase
FPG: Fasting plasma glucose
GGT: Gamma-glutamyl transferase
HbA1c: Hemoglobin A1c
ALT: Alanine aminotransferase
HDL-C: High-density lipoprotein cholesterol
TC: Total cholesterol
OR: Odds ratios
CI: Confidence intervals
NTNW: Normal TG and waist circumference
ETNW: Elevated TG and normal waist circumference
NTEW: Normal TG and enlarged waist circumference
SD: Standard deviation
TGW: Triglyceride waist circumference
