# Supplementary material for: Association between hypertriglyceridemic-waist phenotype and non-alcoholic fatty liver disease: a general population-based study
Source: Lipids Health Dis. 2022 Jun 2;21:50. doi: 10.1186/s12944-022-01660-8 (PMC9161496; doi:10.1186/s12944-022-01660-8)
Supplement: Supplementary file 1 — Additional file 1. [file 12944_2022_1660_MOESM1_ESM.docx]

Supplementary Table 1: Collinearity diagnostics steps.

|  | Variance inflation factor | | | |
| --- | --- | --- | --- | --- |
|  | Step 1 | Step 2 | Step 3 | Step 4 |
| TGW phenotypes | 2.1 | 2.1 | 1.9 | 1.9 |
| Sex | 3.6 | 3.6 | 3.5 | 3.5 |
| Age | 1.4 | 1.4 | 1.3 | 1.3 |
| Height | 51.9 | 2.9 | 2.4 | 2.4 |
| Weight | 168.6 | NA | NA | NA |
| BMI | 95.9 | 5 | 2.2 | 2.2 |
| WC | 6.7 | 6.7 | NA | NA |
| ALT | 4.1 | 4.1 | 4.1 | 4.1 |
| AST | 3.3 | 3.3 | 3.3 | 3.3 |
| GGT | 1.5 | 1.5 | 1.5 | 1.5 |
| HDL-C | 1.8 | 1.8 | 1.8 | 1.8 |
| TC | 1.5 | 1.5 | 1.5 | 1.5 |
| TG | 1.9 | 1.9 | 1.9 | 1.9 |
| FPG | 1.5 | 1.5 | 1.5 | 1.5 |
| HbA1c | 1.2 | 1.2 | 1.2 | 1.2 |
| Habit of exercise | 1 | 1 | 1 | 1 |
| Drinking status | 1.2 | 1.2 | 1.2 | 1.2 |
| Smoking status | 1.4 | 1.4 | 1.4 | 1.4 |
| SBP | 5.5 | 5.5 | 5.5 | 1.4 |
| DBP | 5.6 | 5.6 | 5.6 | NA |

VIF = 1/(1-R^2^). Abbreviations as in Table ​2.
